# Sexuelle Ablehnungskompetenz als Schlüsselfaktor für die sexuelle Gesundheit von trans und nicht-binären Menschen in Deutschland – Quantitative Ergebnisse einer partizipativen Querschnittsbefragung

**DOI:** 10.1007/s00103-026-04216-8

**Published:** 2026-03-13

**Authors:** Kathleen Pöge, Michael Brandl, Manuel Ricardo Garcia, Alexander Hahne, Jonas Hamm, Silvia Rentzsch, Christoph Schuler, Chris Spurgat, Uwe Koppe, TASG study group, Né Fink, Heinz-Jürgen Voss

**Affiliations:** 1 Abteilung 2 Epidemiologie und Gesundheitsmonitoring, Robert Koch-Institut, Berlin, Deutschland Gerichtstraße 27, 13347 Berlin; 2 Abteilung 3 Infektionsepidemiologie, FG 34 – HIV/AIDS und andere sexuell oder durch Blut übertragbare Infektionen, Robert Koch-Institut, Berlin, Deutschland; 3 München, Deutschland; 4 Hamburg, Deutschland; 5 Deutsche Aidshilfe e. V., Berlin, Deutschland; 6 Trans-Inter-Aktiv in Mitteldeutschland e. V., Zwickau, Deutschland; 7 Allgemeinmedizin Praxis Turmstraße, Berlin, Deutschland

**Keywords:** Sexuelle Gesundheit, Sexuelle Kommunikation, Sexuelle Assertivität, Geschlechtliche Minderheiten, Geschlechtliche Diversität, Sexual health, Sexual communication, Sexual assertiveness, Gender minorities, Gender diversity

## Abstract

**Einleitung:**

Sexuelle Ablehnungskompetenz – die Fähigkeit, unerwünschte sexuelle Handlungen abzulehnen – ist zentral für sexuelle Gesundheit und Selbstbestimmung. Für trans und nicht-binäre Menschen in Deutschland liegen bislang kaum quantitative Daten vor.

**Methoden:**

Grundlage ist die partizipative Querschnittsstudie „Sexuelle Gesundheit in trans und nicht-binären Communitys“ (TASG). Die Online-Befragung erfolgte von März bis Juli 2022. In der hier durchgeführten Auswertung wurden in Deutschland lebende, mindestens 18-jährige, sexuell aktive Personen, die sich als trans und/oder nicht-binär identifizierten, eingeschlossen. Es erfolgte eine quantitative Auswertung.

**Ergebnisse:**

Von den insgesamt 1421 Teilnehmenden stimmten 67,7 % „eher“ bis „voll“ der Aussage zu, dass es ihnen leichtfalle, „Nein“ zu unerwünschtem Sex zu sagen (hohe Ablehnungskompetenz). Diese Personen berichteten häufiger eine höhere sexuelle Zufriedenheit. Darüber hinaus war eine hohe Ablehnungskompetenz mit affirmativen, d. h. die eigene geschlechtliche Identität bestätigenden und unterstützenden Lebensbedingungen assoziiert – etwa dem Leben im Identitätsgeschlecht, Zufriedenheit mit dem eigenen Körper, Respekt gegenüber der eigenen geschlechtlichen Identität und sozialer Einbindung. Negative Zusammenhänge zeigten sich mit depressiven und Angstsymptomen, internalisierter Transnegativität, Gewalterfahrungen, Zurückweisungen durch Sexualpartner*innen und dem Gefühl, die eigene geschlechtliche Identität beweisen zu müssen.

**Diskussion:**

Sexuelle Ablehnungskompetenz ist nicht nur individuell bedingt, sondern mit sozialen, körperbezogenen und psychischen Faktoren verknüpft. Gesundheitsförderung und Sexualpädagogik sollten daher trans- und nicht-binäraffirmative sowie partizipative Ansätze verfolgen.

## Abstract (en)

**Introduction:**

Sexual refusal competence—the ability to refuse unwanted sexual acts—is central to sexual health and self-determination. To date, there has been little quantitative data available on trans and non-binary people in Germany.

**Methods:**

The basis for this study is the participatory cross-sectional study “Sexual Health in Trans and Non-Binary Communities” (TASG). The online survey was conducted from March to July 2022. The analysis presented here included sexually active individuals living in Germany who were at least 18 years old and identified as trans and/or non-binary. A quantitative analysis was conducted.

**Results:**

Of the 1421 participants, 67.7% agreed “somewhat” to “completely” with the statement that they found it easy to say “no” to unwanted sex (high level of refusal competence). These individuals reported higher sexual satisfaction more frequently. In addition, high refusal competence was associated with affirmative living conditions, that is, conditions that confirm and support one’s own gender identity, such as living in one’s gender identity, satisfaction with one’s own body, respect for one’s own gender identity, and social inclusion. Negative correlations were found with depressive and anxiety symptoms, internalized transnegativity, experiences of violence, rejection by sexual partners, and the feeling of having to prove one’s gender identity.

**Discussion:**

Sexual rejection competence is not only individually determined, but also linked to social, physical, and psychological factors. Health promotion and sex education should therefore pursue trans- and non-binary-affirmative as well as participatory approaches.

## Einleitung

Sexuelle Gesundheit ist ein zentraler Bestandteil des allgemeinen Wohlbefindens und der Lebensqualität. Die Weltgesundheitsorganisation (WHO) versteht sie als einen Zustand des körperlichen, emotionalen, mentalen und sozialen Wohlbefindens in Bezug auf Sexualität – nicht nur als Abwesenheit von Krankheit oder Funktionsstörung. Sie betont, dass sexuelle Gesundheit auf Achtung, gegenseitiger Zustimmung, Lust und Sicherheit basieren muss, frei von Zwang, Diskriminierung und Gewalt [1].

Ein wesentlicher Aspekt sexueller Gesundheit ist die Fähigkeit, eigene sexuelle Bedürfnisse, Wünsche und Grenzen zu kennen, zu kommunizieren und durchzusetzen. Diese Fähigkeit wird häufig als „sexuelle Verhandlungskompetenz“ oder „sexuelle Assertivität“ bezeichnet und umfasst sowohl das Äußern sexueller Wünsche als auch die Kompetenz, unerwünschte sexuelle Handlungen abzulehnen [2, 3]. Als Teilaspekt der sexuellen Verhandlungskompetenz steht im vorliegenden Beitrag die sexuelle Ablehnungskompetenz von unerwünschten sexuellen Handlungen im Fokus. Eine hohe sexuelle Verhandlungskompetenz ist mit höherer sexueller Zufriedenheit, weniger sexuellen Grenzverletzungen und einem verbesserten Schutz vor sexuell übertragbaren Infektionen (STI), einschließlich HIV, assoziiert [4].

Forschung zur sexuellen Gesundheit von trans und nicht-binären Menschen hat in den letzten Jahren zugenommen, bleibt jedoch häufig auf medizinische und risikoorientierte Perspektiven beschränkt – insbesondere auf HIV/STI-Prävention, sexuelle Dysfunktionen, reproduktive Gesundheit oder Versorgungsbarrieren [5]. Selten und eher in qualitativen Studien wird hingegen die Zufriedenheit mit dem Sexualleben beleuchtet [6]. Dabei zeigen insbesondere qualitative Studien, dass Sexualität für viele trans und nicht-binäre Menschen ein zentraler Bereich von Identität, Selbstwirksamkeit und sozialer Zugehörigkeit ist [7, 8]. Gleichzeitig berichten trans und nicht-binäre Menschen von Diskriminierung, transfeindlicher Gewalt und dem Mangel an affirmativer, d. h. die eigene geschlechtliche Identität bestätigender und unterstützender Gesundheitsversorgung [7, 9, 10].

Im sexuellen Kontext sind trans und nicht-binäre Menschen mit verschiedenen Herausforderungen konfrontiert. Ein zentraler Faktor ist die gesellschaftliche cis- und zweigeschlechtliche Norm. Sie beruht auf der Annahme, dass es nur zwei Geschlechter gibt und dass das bei der Geburt zugeschriebene Geschlecht mit der geschlechtlichen Identität übereinstimmt [11, 12]. Das „Gender Minority Stress and Resilience (GMSR) Measure“ (Messinstrument für Stress und Resilienz bei geschlechtlichen Minderheiten zur Erfassung von Minderheitenstress; [13]) kann herangezogen werden, um zu beschreiben, wie chronischer Stress aufgrund von Stigmatisierung und Diskriminierung das psychische Wohlbefinden und damit auch sexuelle Kompetenzen beeinflussen kann. Verschiedene Studien zeigen, dass internalisierte Transnegativität (d. h. die Verinnerlichung gesellschaftlich abwertender Einstellungen gegenüber trans und nicht-binären Menschen), Geschlechtsdysphorie (d. h. belastende Inkongruenz zwischen geschlechtlicher Identität und bei Geburt zugewiesenem Geschlecht) und mangelnde Affirmation durch Sexualpartner*innen mit geringerer sexueller Zufriedenheit und weniger assertivem Verhalten einhergehen [8, 10, 14]. So zeigte eine qualitative Interviewstudie, dass das sexuelle Erleben von trans Personen, denen bei Geburt das weibliche Geschlecht zugewiesen wurde (Assigned Female at Birth, AFAB), davon beeinflusst wird, inwieweit sie sich von ihren Sexualpartner*innen in ihrer geschlechtlichen Identität akzeptiert und sich mit ihnen sicher fühlen [8]. Auch können internalisierte Transnegativität und Geschlechtsdysphorie die sexuelle Ablehnungskompetenz von trans und nicht-binären Menschen negativ beeinflussen [14–16]. Bei trans und nicht-binären Personen wurde in der internationalen Forschung häufig verringerte Selbstwirksamkeit beim Aushandeln von Schutzmaßnahmen und Grenzen beobachtet; diese reduzierte Verhandlungskompetenz wird wiederholt mit minderheitenbezogenen Stressfaktoren wie Misgendering (d. h. Bezeichnen einer Person mit einem Geschlecht, dass nicht ihrer geschlechtlichen Identität entspricht), „Passing“-Sorgen (Befürchtung, nicht als dem eigenen Geschlecht zugehörig wahrgenommen bzw. erkannt zu werden) und erlebter Diskriminierung in Verbindung gebracht [17, 18].

Obwohl sexuelle Ablehnungskompetenz als wichtiger Schutzfaktor vor sexueller Gewalt in der allgemeinen Sexualforschung etabliert ist, wurde sie in quantitativen Studien zu trans und nicht-binären Menschen bislang kaum untersucht [19, 20]. Bisherige Befunde stammen vor allem aus kleineren qualitativen Arbeiten, die aufzeigen, dass die Möglichkeit, Grenzen zu kommunizieren, eng mit sozialer Unterstützung, Körperzufriedenheit und affirmativen Beziehungserfahrungen verknüpft ist [6, 8]. Damit besteht eine deutliche Forschungslücke hinsichtlich der quantitativen Erfassung sexueller Ablehnungskompetenz bei trans und nicht-binären Menschen.

Vor diesem Hintergrund untersucht der vorliegende Beitrag, wie sexuell aktive trans und nicht-binäre Menschen in Deutschland ihre sexuelle Ablehnungskompetenz einschätzen, wie zufrieden sie mit ihrem Sexualleben sind und welche Subgruppen eine besonders hohe oder niedrige Ablehnungskompetenz berichten. Ziel ist es, differenzierte Einblicke in die Determinanten sexueller Ablehnungskompetenz zu gewinnen und Ansatzpunkte für sexualpädagogische und gesundheitspolitische Interventionen zu identifizieren, die zur Förderung sexueller Gesundheit von trans und nicht-binären Menschen beitragen können.

## Methoden

### Studiendesign und Stichprobe

Die TASG-Studie („Sexuelle Gesundheit in trans und nicht-binären Communitys“) war ein partizipatives Forschungsprojekt zu HIV/STI und sexueller Gesundheit bei trans und nicht-binären Personen in Deutschland, das in Zusammenarbeit mit Mitgliedern der Communitys entwickelt und durchgeführt wurde [10]. Die Studie schloss eine anonyme Online-Befragung ein, die in einem partizipativen Prozess mit Mitgliedern der Communitys erstellt wurde. Die Befragung umfasste Themen im Zusammenhang mit der sexuellen Gesundheit von trans und/oder nicht-binären Personen sowie Einflussfaktoren. Die Rekrutierung fand vom 01.03. bis zum 01.07.2022 statt. Die Personen mussten der Teilnahme an der Befragung zustimmen, sich als Teil des trans und/oder nicht-binären Spektrums identifizieren, ≥ 18 Jahre alt sein und zum Zeitpunkt der Teilnahme in Deutschland wohnen.

Während der Rekrutierung über soziale Medien wurde zwischen dem 28.03. und dem 01.04.2022 ein unerwartet starker Anstieg der Beteiligung beobachtet. Dies wurde auf transfeindliche Reaktionen nach einem Social-Media-Post zurückgeführt, die sich in einigen Freitextkommentaren zur Umfrage widerspiegelten. Die Befragung wurde auch von einigen Personen genutzt, um eine feindselige Haltung gegenüber trans und nicht-binären Menschen zum Ausdruck zu bringen. Die Verfahren zur Datenbereinigung, mit denen irrelevante oder feindselige Beiträge entfernt wurden, werden an anderer Stelle ausführlich beschrieben [10, 21].

Im umfangreichen Forschungsbericht zur TASG-Studie wurden bereits erste Ergebnisse veröffentlicht [21]. In der vorliegenden Arbeit wird die sexuelle Ablehnungskompetenz fokussiert. In die Analysen wurden alle Teilnehmenden eingeschlossen, die in der Studie Angaben dazu gemacht haben, ob sie zu unerwünschten sexuellen Handlungen „Nein“ sagen können, und die berichteten, in den vergangenen 12 Monaten sexuelle Interaktion mit Schleimhautkontakt mit mindestens einer Person gehabt zu haben.

### Indikatoren und Operationalisierung

#### Sexuelle Ablehnungskompetenz.

Die Bewertung der sexuellen Ablehnungskompetenz erfolgte anhand der Antworten auf die Frage: „Es fällt mir leicht, ‚Nein‘ zu Sex zu sagen, den ich nicht möchte“, da damit die selbstwahrgenommene Fähigkeit erfasst wird, unerwünschte sexuelle Handlungen und auch deren Umstände (z. B. Partner*in, Ort) abzulehnen. Die Befragten konnten einzelne Aussagen auf einer 5‑stufigen Skala von „stimme gar nicht zu“ bis „stimme voll zu“ beantworten oder keine Angaben machen. Für die Analysen zusammen mit weiteren Aspekten der sexuellen Ablehnungskompetenz wurden die Antworten zusammengefasst zu „stimme gar nicht bis eher nicht zu“, „teils/teils“ und „stimme eher bis voll zu“. Für alle weiteren Analysen wurde die Variable binär zusammengefasst in „niedrig“ für die Werte „teils/teils bis stimme gar nicht zu“ und in „hoch“ für die Werte „stimme eher bis voll zu“.

#### Stratifizierungsvariablen.

Als Stratifizierungsvariablen wurden soziodemografische Angaben herangezogen: Die selbst angegebene geschlechtliche Identität umfasst die Kategorien weibliches Spektrum, männliches Spektrum, nicht-binäres Spektrum, nicht-binäres weibliches Spektrum, nicht-binäres männliches Spektrum und eine Kategorie für Personen, die weitere Begriffe verwenden, die sich nicht in den zuvor genannten Spektren verorten lassen. Das Alter wurde in den Kategorien 18–29, 30–39 und ≥ 40 Jahre analysiert, wobei die ursprünglichen Kategorien 40–49, 50–59 und 60+ Jahre aufgrund der geringen Fallzahlen zusammengefasst wurden. Weitere Stratifizierungsmerkmale umfassten das Bildungsniveau (niedrig = bis zum Hauptschulabschluss, mittel = Fachhochschulabschluss, Abitur und Lehre oder hoch = ab Universitätsabschluss, Techniker*in oder Meister*in), die selbst angegebene finanzielle Situation in Bezug auf das monatliche Einkommen (kein Einkommen; 1–1000 €; 1001–2000 €; 2001–3000 € und eine zusammengefasste Kategorie für Einkommen > 3001 € gebildet aus den Kategorien 3001–4000 €; 4001–5000 €; > 5000 €). Zudem wurde die Größe des Wohnorts erfasst, in dem die Befragten zum Befragungszeitpunkt lebten (< 20.000; ≥ 20.000 bis 1 Mio.; > 1 Mio. Einwohner*innen sowie „wechselnde Orte“).

#### Körperliches und psychisches Wohlbefinden.

Zu den potenziellen Einflussfaktoren für sexuelle Ablehnungskompetenz gehören Aspekte des körperlichen Wohlbefindens wie Leben entsprechend der geschlechtlichen Identität im täglichen Leben (nein, ja oder teilweise) und die Zufriedenheit mit dem eigenen Körper (sehr zufrieden und etwas zufrieden als „ja“ kodiert; weder zufrieden noch unzufrieden, etwas unzufrieden und sehr unzufrieden als „nein“ kodiert). Zu den Aspekten des psychischen Wohlbefindens zählt die internalisierte Transphobie (hier Transnegativität genannt) und Stolz (*Pride*, hier Transpositivität genannt), welche anhand der jeweiligen Summenscores des Gender Minority Stress and Resilience Measure [13, 22] bewertet wurden. Teilnehmende, die eine Punktzahl zwischen 0 und 16 erreichten, wurden als „neutral bis ablehnend“ eingestuft, diejenigen mit einer Punktzahl zwischen 17 und 32 als „affirmativ“. Depressive Symptome wurden mit dem PHQ-9-Fragebogen [23, 24] gemessen, der für eine geschlechtergerechte Sprache leicht modifiziert wurde. Angstsymptome wurden mit dem GAD-7-Fragebogen [25, 26] erfasst. Die Teilnehmenden wurden anhand ihrer Werte in die Kategorien „keine bis milde Symptome“ (Wert bis 9), „mittlere Symptome“ (Wert 10 bis 14) und „schwere Symptome“ (Wert ≥ 15) eingestuft.

#### Soziale Aspekte.

Im Hinblick auf soziale Aspekte wurde erfasst, ob die eigene geschlechtliche Identität im täglichen Leben respektiert wird (nie; manchmal; meistens oder immer), welcher Beziehungsstatus vorliegt (Single; feste*n Partner*in; mehrere feste Partner*innen/polyamor; eine/mehrere lose Partnerschaft/en; nicht sicher; kompliziert) sowie die Zahl der engen sozialen Kontakte in Gruppen von 0, 1–2 und ≥ 3 ist.

#### Sexueller Kontext.

In die Analysen wurden weiterhin Aspekte des sexuellen Kontexts einbezogen: die Zahl der Sexualpartner*innen in den letzten 12 Monaten (1; 2–3; ≥ 4), sich sexuell begehrt fühlen (stimme gar nicht bis eher nicht zu; teils/teils; stimme eher bis voll zu), Gefühl geschlechtliche Identität mit eigenem Verhalten beweisen zu müssen (nein; ja), Erfahrung, als Sexualpartner*in zurückgewiesen worden zu sein (nein; ja), sexuelle Handlungen wurden gegen den eigenen Willen vorgenommen (nein; ja), Sex war unerwartet gewaltvoll (nein; ja). Weiterhin wurde erfragt, ob die Teilnehmenden Rassismus im sexuellen Kontext erfahren haben: „Ich habe Rassismus erfahren (Ablehnung aufgrund meiner Hautfarbe, meiner Gesichtszüge, meiner Körperformen, meiner Haare, meiner Sprache, meiner Herkunft und damit verbundener Stereotypen)“ (nein; ja).

#### Weitere Kontextfaktoren.

Zu den weiteren Kontextfaktoren, die einen Einfluss auf die sexuelle Ablehnungskompetenz haben können, gehören Wohnungslosigkeit (nein; ja, aktuell; ja, in der Vergangenheit) und das Anbieten von Sexarbeit (nein; ja).

#### Zufriedenheit mit dem Sexualleben.

Die Outcomevariable zur Messung eines wichtigen Aspekts der sexuellen Gesundheit, die selbsteingeschätzte Zufriedenheit mit dem Sexualleben, wurde über die Frage: „Auf einer Skala von 1 bis 10 (1: maximal unzufrieden; 10: maximal zufrieden), wie zufrieden sind Sie mit Ihrem Sexualleben?“, erhoben, wobei der untere Bereich mit 1–3, der mittlere mit 4–7 und der obere mit 8–10 kodiert wurde.

Bei allen oben beschriebenen Variablen wurden Antworten wie „Ich weiß nicht“, „Keine Antwort“ oder das Überspringen der Frage als „missing“ kodiert.

### Statistische Methoden

Die Daten wurden mit deskriptiven Methoden analysiert, wobei für alle kategorialen Variablen absolute und relative Anteile angegeben wurden. Bei den relativen Anteilen (in %) wurden Teilnehmende mit fehlenden Werten für die jeweilige Variable nicht berücksichtigt, es sei denn, die Kategorie „missing“ oder „keine Angabe“ wurde ausdrücklich in die Berechnung des Prozentsatzes einbezogen. Prevalence Ratios (PR) und 95 %-Konfidenzintervalle (KI) wurden anhand eines Poisson-Modells mit robusten Standardfehlern berechnet und zur Untersuchung univariabler Zusammenhänge verwendet. Die Prävalenzverhältnisse wurden als Forest Plots dargestellt. Für Trendanalysen wurde ein χ^2^-Test für Trend durchgeführt.

## Ergebnisse

### Beschreibung der Stichprobe

Insgesamt gaben von den 3077 Studienteilnehmenden 1421 Befragte an, dass sie in den vergangenen 12 Monaten mit mindestens einer Person sexuellen Kontakt hatten, bei dem es zu Berührungen von Schleimhäuten kam, und machten zugleich Angaben zur sexuellen Ablehnungskompetenz. 806 Personen (56,7 %) berichteten von sexuellen Kontakten in den letzten 12 Monaten mit einer Person, 399 (28,1 %) mit 2–3 Personen und 216 (15,2 %) mit 4 oder mehr Personen.

In der Stichprobe sind trans und nicht-binäre Personen enthalten, die sich innerhalb verschiedener Spektren geschlechtlicher Identitäten verorten, wobei mit 30,5 % das nicht-binäre Spektrum am häufigsten vertreten ist (Tab. 1). Mehr als die Hälfte der Befragten (62,0 %) war zwischen 18 und 29 Jahren alt. Mittlere und hohe Bildungsabschlüsse (36,9 % bzw. 32,7 %) waren am häufigsten vertreten. Hinsichtlich des monatlichen Einkommens gaben 33,4 % der Befragten an, über bis zu 1000 € zu verfügen; 25,3 % verfügten über 1001 bis 2000 €. Über die Hälfte der Befragten lebte in einer Stadt mit 20.000 bis 1 Mio. Einwohner*innen, 27,9 % in einer Metropole über 1 Mio. Einwohner*innen.

**Tab. 1:** Beschreibung der Stichprobe nach soziodemografischen Angaben (*n* = 1421)

Merkmal	Ausprägung	Anteil^a^
Geschlechtliche Identität	Weibliches Spektrum	261 (18,4 %)
	Männliches Spektrum	313 (22,0 %)
	Nicht-binäres Spektrum	434 (30,5 %)
	Nicht-binäres weibliches Spektrum	180 (12,7 %)
	Nicht-binäres männliches Spektrum	218 (15,3 %)
	Weitere Bezeichnung	15 (1,1 %)
Alter	18–29 Jahre	881 (62,0 %)
	30–39 Jahre	374 (26,3 %)
	40+ Jahre	166 (11,7 %)
Bildung	Niedrig	138 (9,7 %)
	Mittel	525 (36,9 %)
	Hoch	464 (32,7 %)
	Keine Angabe	294 (20,7 %)
Einkommen	Kein Einkommen	48 (3,4 %)
	1–1000 €	474 (33,4 %)
	1001–2000 €	359 (25,3 %)
	2001–3000 €	153 (10,8 %)
	3001–4000 €	41 (2,9 %)
	4001–5000 €	37 (2,6 %)
	Keine Angabe	309 (21,7 %)
Wohnortgröße (Zahl der Einwohner*innen)	Metropole > 1 Mio.	397 (27,9 %)
	Stadt 20.000–1 Mio.	771 (54,3 %)
	Ländlich bis 20.000	226 (15,9 %)
	Wechselnde Orte	22 (1,5 %)
	Keine Angabe	5 (0,4 %)
Sexuelle Ablehnungskompetenz (Zustimmung zur Frage: „Es fällt mir leicht, ‚Nein‘ zu Sex zu sagen, den ich nicht möchte“)	Stimme gar nicht zu	56 (3,9 %)
	Stimme eher nicht zu	183 (12,9 %)
	Teil, teils	220 (15,5 %)
	Stimme eher zu	432 (30,4 %)
	Stimme voll zu	530 (37,3 %)

^a^ Die Prozentwerte können sich aufgrund der Rundung nicht exakt zu 100 % addieren

### Verteilung von sexueller Zufriedenheit nach Ablehnungskompetenz

Zwei Drittel der Befragten (962, 67,7 %) stimmten der Aussage voll bis eher zu, dass es ihnen leichtfalle, zu Sex „Nein“ zu sagen, den sie nicht wollten, was als hohe sexuelle Ablehnungskompetenz definiert wurde. 220 Personen (15,5 %) stimmten der Aussage teilweise zu und 239 Personen (16,8 %) stimmten eher bis gar nicht zu (Tab. 1). Es zeigte sich, dass Personen mit einer hohen Ablehnungskompetenz meist eine Zufriedenheit mit ihrem Sexualleben im hohen (354, 36,8 %) oder mittleren Bereich (454, 47,2 %) berichteten (vs. 134, 13,9 % unterer Bereich; Tab. 2). Personen mit geringer Ablehnungskompetenz gaben vergleichsweise häufiger an, weniger zufrieden mit ihrem Sexualleben zu sein (oberer und mittlerer Bereich: 46 (19,2 %) und 117 (49,0 %) vs. unterer Bereich 70 (29,3 %)).

**Tab. 2:** Assoziation von sexueller Ablehnungskompetenz und Zufriedenheit mit dem Sexualleben (*n* = 1421)

Zufriedenheit mit Sexualleben	Fällt leicht, „Nein“ zu sagen zu Sex, den ich nicht möchte		
	Stimme gar nicht bis eher nicht zu (*n* = 239)	Teils, teils (*n* = 220)	Stimme eher bis voll zu (*n* = 962)
Unterer Bereich	70 (29,3 %)	40 (18,2 %)	134 (13,9 %)
Mittlerer Bereich	117 (49,0 %)	122 (55,5 %)	454 (47,2 %)
Oberer Bereich	46 (19,2 %)	55 (25,0 %)	354 (36,8 %)
Keine Angabe	6 (2,5 %)	3 (1,4 %)	20 (2,1 %)

Die Prozentwerte können sich aufgrund der Rundung nicht exakt zu 100 % addieren

### Sexuelle Ablehnungskompetenz und ihre Determinanten

**Abb. 1:**
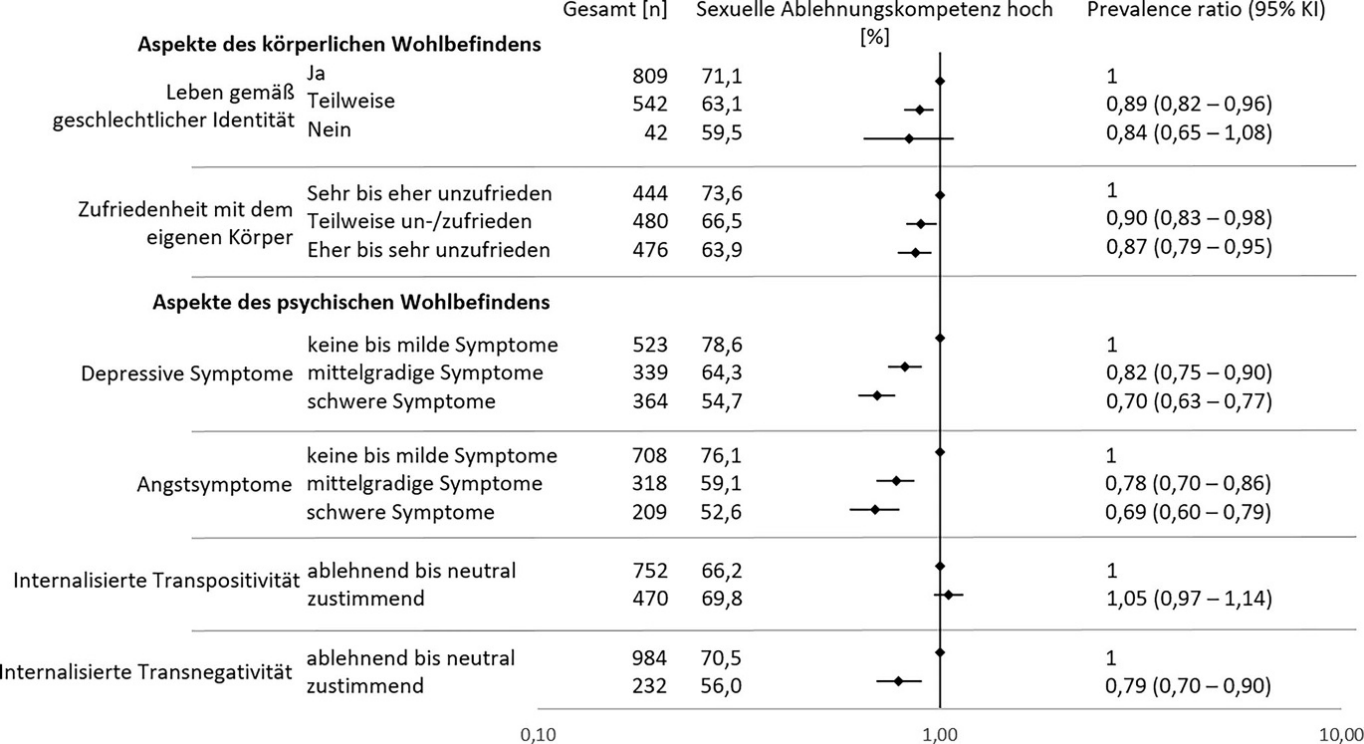
Prävalenzverhältnisse (Prevalence Ratios) für eine hohe sexuelle Ablehnungskompetenz nach Aspekten des körperlichen und psychischen Wohlbefindens. Die Effektschätzer sind durch Rauten gekennzeichnet. Effektschätzer < 1 weisen auf eine niedrigere Prävalenz von hoher sexueller Ablehnungskompetenz im Vergleich zur Referenz hin, während Effektschätzer > 1 eine höhere Prävalenz anzeigen. *KI* Konfidenzintervall

Im Hinblick auf das körperliche Wohlbefinden waren sowohl das Leben entsprechend der geschlechtlichen Identität als auch die Zufriedenheit mit dem eigenen Körper mit einer hohen Ablehnungskompetenz assoziiert (Abb. 1; Tab. 3). Im Vergleich zu Personen, die entsprechend ihrer geschlechtlichen Identität lebten, wiesen Personen, die dies nur teilweise (PR 0,89, 95 %-KI 0,82–0,96) oder nicht taten (PR 0,84, 95 %-KI 0,65–1,08) niedrigere Anteile hoher Ablehnungskompetenz auf. Aufgrund der geringen Fallzahl (*n* = 42) sind jedoch die Ergebnisse zu Personen, die bisher nicht entsprechend ihrer geschlechtlichen Identität gelebt haben, nur eingeschränkt aussagekräftig. Weiterhin wiesen Befragte, die mit dem eigenen Körper zufriedener waren, mit 73,6 % häufiger eine hohe Ablehnungskompetenz auf, im Vergleich zu Personen, die teilweise (66,5 %, PR 0,90, 95 %-KI 0,83–0,98) oder eher bis sehr unzufrieden waren (63,9 %, PR 0,87, 95 %-KI 0,79–0,95).

**Tab. 3:** Assoziation von sexueller Ablehnungskompetenz mit verschiedenen Aspekten des körperlichen und psychischen Wohlbefindens sowie sozialen, sexuellen und weiteren Aspekten

		Sexuelle Ablehnungskompetenz	
		Niedrig	Hoch
*Aspekte des körperlichen Wohlbefindens*	*Leben gemäß geschlechtlicher Identität*		
	Ja	234 (28,9 %)	575 (71,1 %)
	Teilweise	200 (36,9 %)	342 (63,1 %)
	Nein	17 (40,5 %)	25 (59,5 %)
	Keine Angabe	8 (28,6 %)	20 (71,4 %)
	*Zufriedenheit mit dem eigenen Körper*		
	Sehr bis eher zufrieden	117 (26,4 %)	327 (73,6 %)
	Teilweise un-/zufrieden	161 (33,5 %)	319 (66,5 %)
	Eher bis sehr unzufrieden	172 (36,1 %)	304 (63,9 %)
	Keine Angabe	9 (42,9 %)	12 (57,1 %)
*Aspekte des psychischen Wohlbefindens*	*Depressive Symptome*		
	Keine bis milde Symptome	112 (21,4 %)	411 (78,6 %)
	Mittelgradige Symptome	121 (35,7 %)	218 (64,3 %)
	Schwer	165 (45,3 %)	199 (54,7 %)
	Keine Angabe	61 (31,3 %)	134 (68,7 %)
	*Angstsymptome*		
	Keine bis milde Symptome	169 (23,9 %)	539 (76,1 %)
	Mittelgradige Symptome	130 (40,9 %)	188 (59,1 %)
	Schwer	99 (47,4 %)	110 (52,6 %)
	Keine Angabe	61 (32,8 %)	125 (67,2 %)
	*Internalisierte Transpositivität*		
	Ablehnend bis neutral	254 (33,8 %)	498 (66,2 %)
	Zustimmend	142 (30,2 %)	328 (69,8 %)
	Keine Angabe	63 (31,7 %)	136 (68,3 %)
	*Internalisierte Transnegativität*		
	Ablehnend bis neutral	290 (29,5 %)	694 (70,5 %)
	Zustimmend	102 (44,0 %)	130 (56,0 %)
	Keine Angabe	67 (32,7 %)	138 (67,3 %)
*Soziale Aspekte*	*Identität im Alltag respektiert*		
	Nie	13 (44,8 %)	16 (55,2 %)
	Manchmal	189 (37,1 %)	320 (62,9 %)
	Meistens	196 (30,1 %)	455 (69,9 %)
	Immer	44 (26,2 %)	124 (73,8 %)
	Keine Angabe	17 (26,6 %)	47 (73,4 %)
	*Beziehungsstatus*		
	Ich bin Single	123 (40,6 %)	180 (59,4 %)
	Ich habe eine*n festen Partner*in	171 (25,3 %)	505 (74,7 %)
	Ich habe mehrere feste Partner*innen/lebe polyamor	81 (34,3 %)	155 (65,7 %)
	Ich habe eine/mehrere lose Partnerschaft(en)	55 (42,0 %)	76 (58,0 %)
	Ich bin mir nicht sicher	8 (47,1 %)	9 (52,9 %)
	Es ist kompliziert	21 (40,4 %)	31 (59,6 %)
	Keine Angabe	0 (0,0 %)	6 (100,0 %)
	*Zahl enger sozialer Kontakte*		
	Keine	13 (52,0 %)	12 (48,0 %)
	1–2	150 (36,2 %)	264 (63,8 %)
	≥ 3	225 (29,2 %)	546 (70,8 %)
	Keine Angabe	71 (33,6 %)	140 (66,4 %)
*Sexuelle Aspekte*	Partner*innenzahl letzte 12 Monate		
	1 Partner*in	218 (27,0 %)	588 (73,0 %)
	2–3 Partner*innen	156 (39,1 %)	243 (60,9 %)
	4+ Partner*innen	85 (39,4 %)	131 (60,6 %)
	*Sich sexuell begehrt fühlen*		
	Stimme gar nicht bis eher nicht zu	122 (46,0 %)	143 (54,0 %)
	Teils, teils	118 (37,1 %)	200 (62,9 %)
	Stimme eher bis voll zu	211 (25,9 %)	605 (74,1 %)
	Keine Angabe	8 (36,4 %)	14 (63,6 %)
	*Zurückweisung als Sexualpartner erfahren*		
	Nein	106 (25,1 %)	316 (74,9 %)
	Ja	160 (34,4 %)	305 (65,6 %)
	Keine Angabe	193 (36,1 %)	341 (63,9 %)
	*Sexuelle Handlungen wurden gegen den eigenen Willen vorgenommen*		
	Nein	89 (19,9 %)	359 (80,1 %)
	Ja	192 (41,1 %)	275 (58,9 %)
	Keine Angabe	178 (35,2 %)	328 (64,8 %)
	*Gefühl, geschlechtliche Identität mit eigenem Verhalten beweisen zu müssen*		
	Nein	29 (15,5 %)	158 (84,5 %)
	Ja	258 (34,0 %)	500 (66,0 %)
	Keine Angabe	172 (36,1 %)	304 (63,9 %)
	*Sex war unerwartet gewaltvoll*		
	Nein	176 (25,1 %)	526 (74,9 %)
	Ja	101 (47,6 %)	111 (52,4 %)
	Keine Angabe	182 (35,9 %)	325 (64,1 %)
	*Rassismus im sexuellen Kontext erlebt*		
	Nein	260 (30,4 %)	595 (69,6 %)
	Ja	17 (31,5 %)	37 (68,5 %)
	Keine Angabe	182 (35,5 %)	330 (64,5 %)
*Weitere Aspekte*	*Erfahrung mit Wohnungslosigkeit*		
	Nein	314 (31,4 %)	686 (68,6 %)
	Ja, aktuell	7 (63,6 %)	4 (36,4 %)
	Ja, in der Vergangenheit	32 (29,9 %)	75 (70,1 %)
	Keine Angabe	106 (35,0 %)	197 (65,0 %)
	*Anbieten von Sexarbeit*		
	Nein	409 (31,1 %)	906 (68,9 %)
	Ja	41 (52,6 %)	37 (47,4 %)
	Keine Angabe	9 (32,1 %)	19 (67,9 %)

Die Prozentwerte können sich aufgrund der Rundung nicht exakt zu 100 % addieren

Im Bereich des psychischen Wohlbefindens wiesen Personen mit mittelgradigen bis schweren depressiven oder Angstsymptomen seltener eine hohe Ablehnungskompetenz auf. Unter Personen ohne oder mit milden depressiven Symptomen lag der Anteil mit hoher Ablehnungskompetenz bei 78,6 %. Bei mittelgradigen depressiven Symptomen betrug er 64,3 % (PR 0,82, 95 %-KI 0,75–0,90), bei schweren depressiven Symptomen 54,7 % (PR 0,70, 95 %-KI 0,63–0,77). In Bezug auf Angstsymptome zeigte sich ein ähnliches Muster: 76,1 % der Befragten wiesen eine hohe Ablehnungskompetenz auf, verglichen mit 59,1 % bei mittelschweren (PR 0,78, 95 %-KI 0,70–0,86) und 52,6 % bei schweren Angstsymptomen (PR 0,69, 95 %-KI 0,60–0,79). Während eine hohe Ablehnungskompetenz vergleichbar häufig bei Personen mit und ohne internalisierte Transpositivität vorkommt, wiesen Personen, die den Aussagen zu internalisierter Transnegativität häufiger zustimmten, seltener eine hohe Ablehnungskompetenz auf (PR 0,79, 95 %-KI 0,70–0,90).

**Abb. 2:**
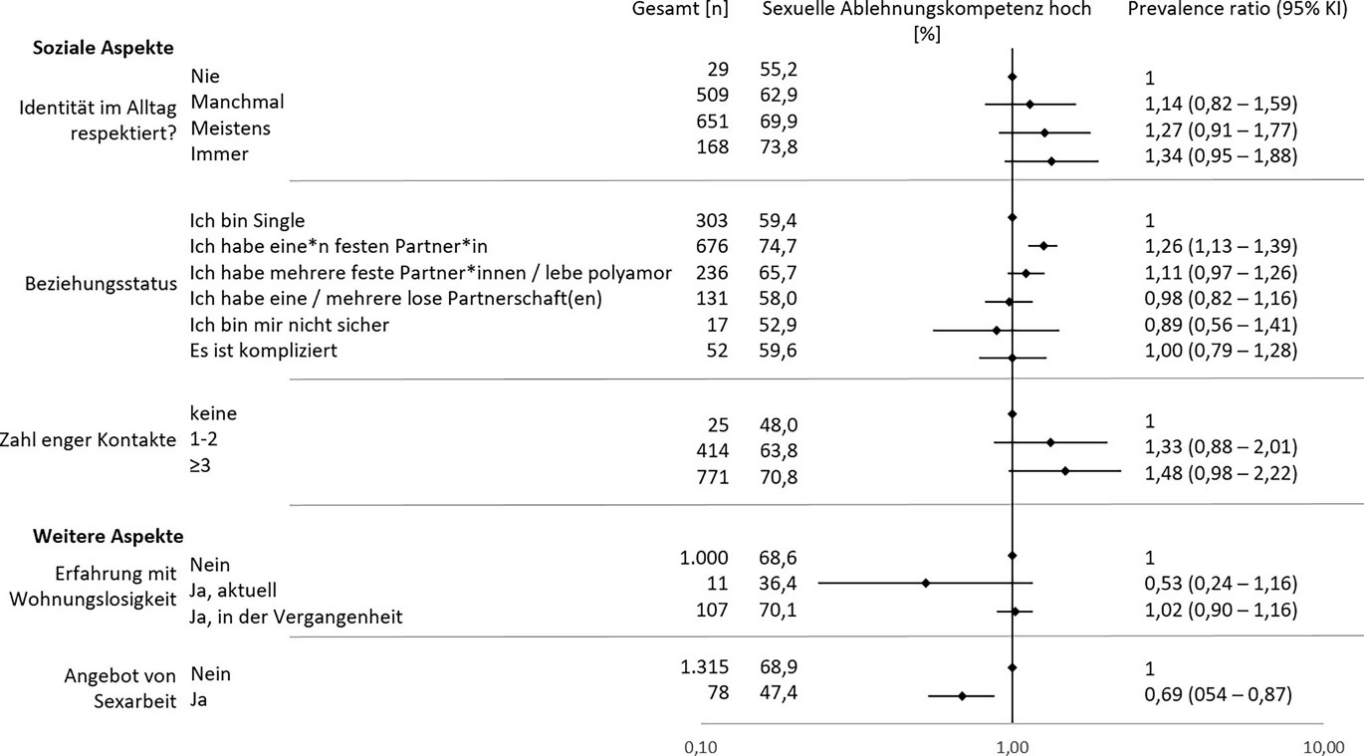
Prävalenzverhältnisse (Prevalence Ratios) für eine hohe sexuelle Ablehnungskompetenz nach sozialen und weiteren Aspekten. *KI* Konfidenzintervall

Im Bereich der sozialen Aspekte war ein Zusammenhang zwischen der berichteten Erfahrung, dass die eigene geschlechtliche Identität im Alltag respektiert wird, und der Ablehnungskompetenz zu beobachten (Abb. 2; Tab. 3). Personen, die angaben, dass ihre geschlechtliche Identität immer (73,8 %, PR 1,34, 95 %-KI 0,95–1,88) oder meistens (69,9 %, PR 1,27, 95 %-KI 0,91–1,77) respektiert wird, wiesen häufiger eine hohe Ablehnungskompetenz auf im Vergleich zu Personen, die angaben, dass ihre geschlechtliche Identität manchmal (62,9 %, PR 1,14, 95 %-KI 0,82–1,59) oder nie (55,2 %) respektiert wird. Zwar sind einige Strata klein und die Konfidenzintervalle der Prevalence Ratios reichen über den Nullwert hinaus, jedoch wurde eine starke Evidenz für einen Trend beobachtet (χ^2^-Test für Trend: *p* < 0,001). In Bezug auf den Beziehungsstatus zeigte sich, dass Personen mit einer*einem festen Partner*in im Vergleich zu allen anderen mit 74,7 % häufiger eine hohe Ablehnungskompetenz angaben. Auch in Bezug auf die soziale Einbindung lagen die Konfidenzintervalle der einzelnen Strata jenseits des Nullwerts. Jedoch konnte auch hier ein positiver Gradient zwischen der Anzahl an engen sozialen Kontakten und der Ablehnungskompetenz beobachtet werden (χ^2^-Test für Trend: *p* = 0,002). So war eine hohe Ablehnungskompetenz bei Personen mit 3 oder mehr engen sozialen Kontakten mit 70,8 % (PR 1,48, 95 %-KI 0,98–2,22) häufiger zu finden als bei Personen mit 1–2 (63,8 %, PR 1,33, 95 %-KI 0,88–2,01) oder keinen engen sozialen Kontakten (48,0 %).

Bei den weiteren Kontextfaktoren zeigt sich, dass Personen, die Sexarbeit anboten, zu 47,4 % eine hohe Ablehnungskompetenz aufwiesen (PR 0,69, 95 %-KI 0,54–0,87) im Vergleich zu 68,9 % der Personen, die keine Sexarbeit anboten. Weiterhin hatten 36,4 % der Befragten mit aktueller Erfahrung von Wohnungslosigkeit deutlich seltener eine hohe Ablehnungskompetenz (PR 0,53, 95 %-KI 0,24–1,16) als Befragte, die keine Erfahrungen mit Wohnungslosigkeit aufwiesen (68,8 %). Aufgrund der geringen Fallzahlen der Teilnehmenden mit aktuellen Erfahrungen von Wohnungslosigkeit (*n* = 11) ist dieses Ergebnis jedoch begrenzt aussagekräftig und könnte auch ein Zufallsbefund sein.

**Abb. 3:**
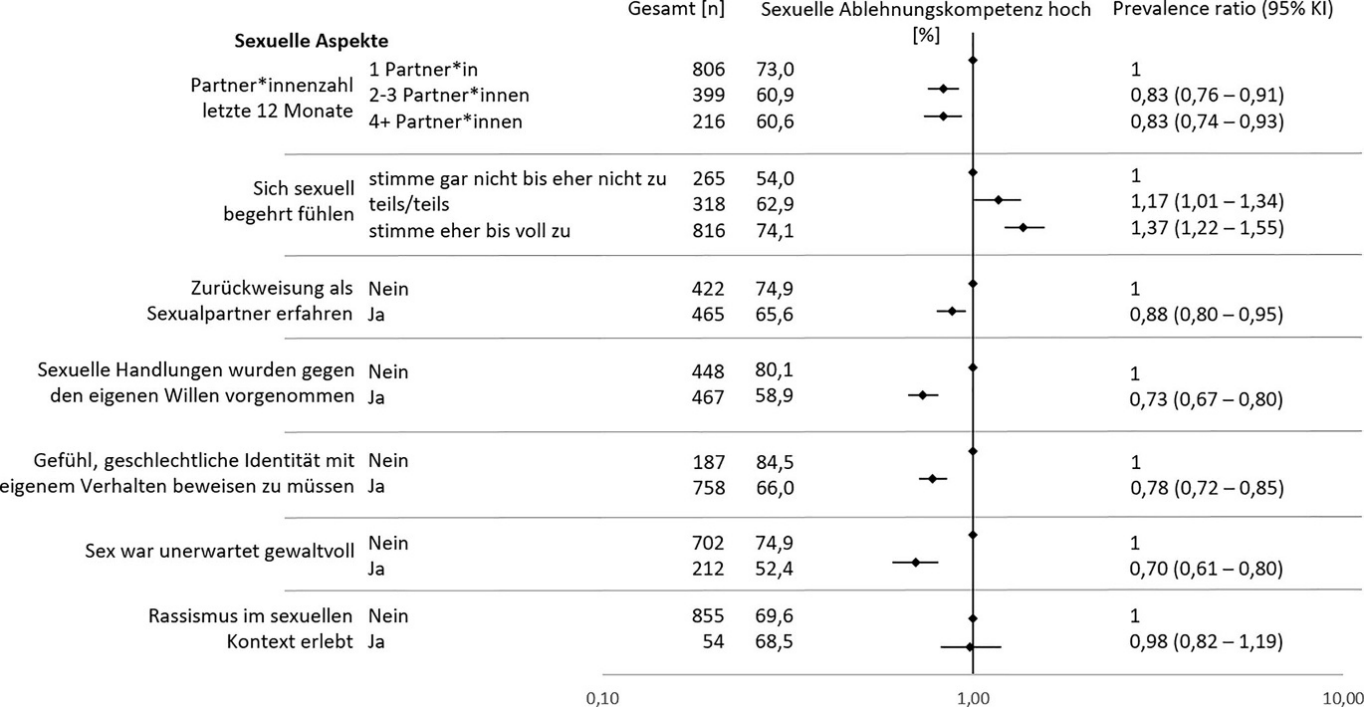
Prevalence Ratios für eine hohe sexuelle Ablehnungskompetenz nach sexuellen Aspekten

Bei folgenden Aspekten des Sexuallebens war häufiger eine hohe Ablehnungskompetenz zu finden: Abwesenheit von Gewalterfahrungen, das Gefühl, sexuell begehrt zu werden, und das Fehlen des Gefühls, die geschlechtliche Identität mit dem eigenen Verhalten beweisen zu müssen (Abb. 3; Tab. 3). So wiesen nur 54,0 % der Personen eine hohe Ablehnungskompetenz auf, die der Aussage eher bis gar nicht zugestimmt hatten, dass sie sich sexuell begehrt fühlten, im Vergleich zu 62,9 % (PR 1,17, 95 %-KI 1,01–1,34) der Personen, die der Aussage teilweise, und 74,1 % (PR 1,37, 95 %-KI 1,22–1,55) der Personen, die voll und ganz zustimmten. Personen, die vom Gefühl berichteten, ihre geschlechtliche Identität in ihrem Verhalten beweisen zu müssen, hatten mit 66,0 % (PR 0,78, 95 %-KI 0,72–85) seltener eine hohe Ablehnungskompetenz als Personen, die nicht von diesem Gefühl berichteten (84,5 %). Ebenfalls seltener kam eine hohe Ablehnungskompetenz bei Personen vor, die von der Erfahrung berichteten, dass sexuelle Handlungen gegen ihren Willen vorgenommen wurden (58,9 %, PR 0,73, 95 %-KI 0,67–0,80 vs. nein: 80,1 %) oder der Sex unerwartet gewaltvoll war (52,4 %, PR 0,70, 95 %-KI 0,61–0,80 vs. nein: 74,9 %). Personen, die Rassismus im sexuellen Kontext erlebt hatten, wiesen eine vergleichbar hohe sexuelle Ablehnungskompetenz auf, wie jene ohne Rassismuserfahrungen. Allerdings ist auch die Gruppe derjenigen, die Rassismus erfahren haben, mit *n* = 54 relativ klein, sodass die Aussagekraft eingeschränkt sein könnte.

## Diskussion

Die vorliegende Studie zeigt, dass sexuelle Ablehnungskompetenz ein zentraler Bestandteil sexueller Gesundheit und sexueller Zufriedenheit von trans und nicht-binären Menschen ist. Etwa zwei Drittel der Befragten gaben an, dass es ihnen „völlig“ oder „eher“ leichtfalle, „Nein“ zu Sex zu sagen, den sie nicht möchten. Dieser Befund verweist darauf, dass viele trans und nicht-binäre Menschen trotz struktureller Diskriminierung über erhebliche Ressourcen der Selbstbestimmung verfügen. Zugleich offenbaren sich deutliche Unterschiede zwischen Subgruppen – insbesondere in Abhängigkeit von psychischen, sozialen und körperbezogenen Aspekten sowie affirmativen Lebensbedingungen. Die Ergebnisse zeigen, dass die sexuelle Ablehnungskompetenz nicht allein eine individuelle Fähigkeit ist, sondern eng mit sozialen Determinanten von Gesundheit verknüpft ist.

Soziale Determinanten, wie das Vorhandensein von engen Sozialkontakten und im Alltag im Identitätsgeschlecht respektiert zu werden, stehen in positiver Korrelation mit Ablehnungskompetenz, ebenso Aspekte des körperlichen Wohlbefindens, wie im Identitätsgeschlecht zu leben und mit dem eigenen Körper zufrieden zu sein. Psychische Belastungen in Form von depressiven und Angstsymptomen sowie Aspekte, wie internalisierte Transnegativität, Unzufriedenheit mit dem eigenen Körper, Zurückweisung als Sexualpartner*in zu erfahren, die Erfahrung von Gewalt und die Wahrnehmung, sich in der geschlechtlichen Identität im Verhalten beweisen zu müssen, korrelieren negativ mit sexueller Ablehnungskompetenz. Sexuelle Zufriedenheit und das Gefühl, sexuell begehrt zu werden, stehen hingegen in einem positiven Zusammenhang mit sexueller Ablehnungskompetenz.

Die Ergebnisse stehen im Einklang mit früheren Untersuchungen, die zeigen, dass sexuelle Assertivität ein Schlüsselfaktor sexueller Zufriedenheit ist [2]. Studien in der allgemeinen Bevölkerung zeigen, dass Personen, die in der Lage sind, sexuelle Grenzen klar zu kommunizieren, häufiger von erfüllter, sicherer und konsensualer Sexualität berichten [3]. Für trans und nicht-binäre Menschen wurde dieser Zusammenhang bislang kaum quantitativ untersucht. Qualitative Arbeiten deuten jedoch darauf hin, dass die Fähigkeit zur Grenzziehung stark vom Erleben geschlechtlicher Affirmation und Akzeptanz abhängt [7, 8].

Unsere Ergebnisse bestätigen und erweitern diese Befunde. Sie zeigen, dass die sexuelle Ablehnungskompetenz hoch ist, wenn Menschen entsprechend ihrer geschlechtlichen Identität leben können, sich mit ihrem Körper wohlfühlen und soziale Unterstützung erfahren. Diese Befunde stehen in Übereinstimmung mit dem „Gender Minority Stress and Resilience Model“ [13], das betont, dass Resilienzfaktoren wie soziale Einbindung die negativen Effekte von Minderheitenstress abmildern können. Gleichzeitig war jedoch internalisierte Transpositivität nicht mit einer höheren Ablehnungskompetenz verbunden, während internalisierte Transnegativität negativ korreliert war: Personen, die negative gesellschaftliche Einstellungen internalisiert hatten, berichteten seltener von einer hohen Ablehnungskompetenz. Die im GMSR Model benannten Aspekte erweisen sich in der vorliegenden Studie als unterschiedlich gewichtet in ihrem Einfluss auf die Ablehnungskompetenz.

Unsere Analysen bestätigen die in der Literatur beschriebenen Zusammenhänge zwischen psychischer Belastung und eingeschränkter sexueller Selbstbestimmung. Personen mit mittelgradigen bis schweren depressiven oder Angstsymptomen berichteten deutlich seltener eine hohe Ablehnungskompetenz. Frühere Studien verweisen darauf, dass internalisierte Transnegativität und Geschlechtsdysphorie zentrale Mediatoren zwischen Minderheitenstress und psychischer Gesundheit darstellen [14, 15]. Diese Faktoren können Scham, Unsicherheit und Abhängigkeit in sexuellen Situationen verstärken und damit die Fähigkeit zur Abgrenzung beeinträchtigen.

Körperzufriedenheit und das Leben im eigenen Identitätsgeschlecht gingen mit höheren Anteilen hoher sexueller Ablehnungskompetenz einher. Frühere Arbeiten haben gezeigt, dass Körperakzeptanz eng mit Selbstwirksamkeit und sexueller Lust assoziiert ist [19, 27]. Bei trans und nicht-binären Menschen spielt Körperzufriedenheit zudem eine doppelte Rolle: Sie wirkt nicht nur auf die sexuelle Selbstwahrnehmung, sondern auch auf das Sicherheitsempfinden in intimen Begegnungen [6]. Umgekehrt berichteten Personen, die sich sexuell weniger begehrt fühlten oder Erfahrungen von Zurückweisung, Gewalt oder dem „Beweisen-Müssen“ ihrer geschlechtlichen Identität machten, weniger häufig eine hohe Ablehnungskompetenz. Bisherige Studien konnten z. B. bei cis Frauen zeigen, dass Gewalterfahrungen zu einer geringeren sexuellen Assertivität führen können und dies ein Prädiktor für weitere Gewalterfahrungen war [28]. Diese Befunde verdeutlichen, dass sich sexuelle Handlungsspielräume trans und nicht-binärer Menschen häufig im Spannungsfeld zwischen gesellschaftlicher Marginalisierung und affirmativen Beziehungserfahrungen bewegen.

Zudem zeigte sich, dass das Gefühl, sexuell begehrt zu werden, positiv mit der Fähigkeit zur sexuellen Grenzziehung verknüpft war. Dieses Ergebnis spiegelt Befunde aus der allgemeinen Sexualitätsforschung wider, die ein reziprokes Verhältnis zwischen Selbstwertgefühl, sexueller Attraktivitätswahrnehmung und sexueller Assertivität beschreiben [29]. Trans und nicht-binäre Menschen erleben häufig, dass gesellschaftliche Fremdzuschreibungen ihre sexuelle Selbstwahrnehmung beeinflussen [7]. Affirmative Beziehungserfahrungen, in denen Begehrensfähigkeit unabhängig von cis-normativen Schönheitsidealen anerkannt wird, können daher zentrale Faktoren für sexuelle Selbstbestimmung darstellen.

Ein Ansatzpunkt zur Stärkung der sexuellen Ablehnungskompetenz könnte die Integration inklusiver, trans- und nicht-binäraffirmativer Sexualpädagogik in bestehende Präventions- und Bildungsprogramme sein. Fachkräfte der sexuellen Bildung sollten trans- und nicht-binäraffirmativ arbeiten und ausgebildet werden. Peer-geführte Empowerment-Formate könnten Selbstwirksamkeit und Kommunikationskompetenz stärken. Angebote sollten rassistische und ableistische (auf Menschen mit Behinderungen bzw. Beeinträchtigungen bezogene) Diskriminierungs- und Gewalterfahrungen sensibel berücksichtigen.

Eine Limitation der vorliegenden Studie besteht darin, dass es sich um Befragungsdaten handelt, die erstens bei retrospektiven Angaben abhängig von der Erinnerungsleistung der Befragten sind und zweitens im Fall der sexuellen Ablehnungskompetenz keine direkten Rückschlüsse auf das individuelle Handeln ermöglichen. Allein das Berichten, dass es einer Person schwerfalle, zu ungewollten sexuellen Handlungen „Nein“ zu sagen, bedeutet nicht automatisch, dass in entsprechenden Situationen nicht dennoch unerwünschte sexuelle Handlungen abgelehnt werden. Eine weitere Limitation liegt darin, dass sich anhand von Daten aus einer Querschnittsbefragung keine zeitlich kausalen Zusammenhänge analysieren lassen.

## Fazit

Sexuelle Ablehnungskompetenz von trans und nicht-binären Personen ist nicht nur eine individuelle Fähigkeit, sondern wird von gesellschaftlichen und sozialen Bedingungen geprägt. Zukünftige Forschung sollte intersektionale Perspektiven einbeziehen. Besonders wenig ist bisher über die Situation von trans und nicht-binären Menschen bekannt, die zusätzlich von Rassismus und/oder Armut betroffen sind, eine Behinderung aufweisen oder Sexarbeit nachgehen. Zudem sollten Längsschnittstudien untersuchen, ob und wie Interventionen zur Förderung von sexueller Ablehnungskompetenz langfristig die sexuelle Zufriedenheit und psychische Gesundheit stärken.

Sexuelle Ablehnungskompetenz erweist sich für trans und nicht-binäre Menschen als Schlüsselfaktor sexueller Zufriedenheit und sexueller Selbstbestimmung. Sie ist eng mit psychischen Aspekten, Körperzufriedenheit, sozialer Einbindung und affirmativen Lebensbedingungen verbunden. Eine trans- und nicht-binärinklusive sexuelle Bildung und genderaffirmative Versorgung sind daher zentrale Ansatzpunkte, um sexuelle Handlungsspielräume zu erweitern, Gewalt vorzubeugen und das Recht auf sexuelle Selbstbestimmung für alle Menschen zu verwirklichen.

## Data Availability

Die während der vorliegenden Studie erzeugten und/oder analysierten Datensätze sind aufgrund von Datenschutz nicht öffentlich zugänglich, können aber auf begründete Anfrage von den entsprechenden Autor*innen angefordert werden.
