# Erratum zu: Sexuelle Ablehnungskompetenz als Schlüsselfaktor für die sexuelle Gesundheit von trans und nicht-binären Menschen in Deutschland – Quantitative Ergebnisse einer partizipativen Querschnittsbefragung

**DOI:** 10.1007/s00103-026-04226-6

**Published:** 2026-03-24

**Authors:** Kathleen Pöge, Michael Brandl, Manuel Ricardo Garcia, Alexander Hahne, Jonas Hamm, Silvia Rentzsch, Christoph Schuler, Chris Spurgat, Uwe Koppe, TASG study group, Né Fink, Heinz-Jürgen Voss

**Affiliations:** 1 Abteilung 2 Epidemiologie und Gesundheitsmonitoring, Robert Koch-Institut, Berlin, Deutschland Gerichtstraße 27, 13347; 2 Abteilung 3 Infektionsepidemiologie, FG 34 – HIV/AIDS und andere sexuell oder durch Blut übertragbare Infektionen, Robert Koch-Institut, Berlin, Deutschland; 3 München, Deutschland; 4 Hamburg, Deutschland; 5 Deutsche Aidshilfe e. V., Berlin, Deutschland; 6 Trans-Inter-Aktiv in Mitteldeutschland e. V., Zwickau, Deutschland; 7 Allgemeinmedizin Praxis Turmstraße, Berlin, Deutschland

**Erratum zu:**

**Bundesgesundheitsbl 2026**

10.1007/s00103-026-04216-8

In diesem Artikel hätte der Satz „Personen, die Rassismus im sexuellen Kontext erlebt hatten, wiesen haufiger eine hohe sexuelle Ablehnungskompetenz auf, als jene ohne Rassismuserfahrungen.“ richtigerweise „Personen, die Rassismus im sexuellen Kontext erlebt hatten, wiesen eine vergleichbar hohe sexuelle Ablehnungskompetenz auf, wie jene ohne Rassismuserfahrungen.“ lauten sollen.

Der Originalbeitrag wurde korrigiert.

